# Increasing Internodal Distance in Myelinated Nerves Accelerates Nerve Conduction to a Flat Maximum

**DOI:** 10.1016/j.cub.2012.08.025

**Published:** 2012-10-23

**Authors:** Lai Man N. Wu, Anna Williams, Ada Delaney, Diane L. Sherman, Peter J. Brophy

**Affiliations:** 1 Centre for Neuroregeneration, University of Edinburgh, Edinburgh EH16 4SB, UK; 2 MRC Centre for Regenerative Medicine and Centre for Multiple Sclerosis Research, University of Edinburgh, Edinburgh EH16 4UU, UK

## Abstract

Predictions that conduction velocities are sensitive to the distance between nodes of Ranvier in myelinated axons have implications for nervous system function during growth and repair [1–3]. Internodal lengths defined by Schwann cells in hindlimb nerves, for example, can undergo a 4-fold increase during mouse development, and regenerated nerves have internodes that are uniformly short [4, 5]. Nevertheless, the influence of internodal length on conduction speed has limited experimental support. Here, we examined this problem in mice expressing a mutant version of periaxin, a protein required for Schwann cell elongation [4]. Importantly, elongation of mutant Schwann cells was retarded without significant derangements to myelination or axon caliber. In young mice with short mutant Schwann cells, nerve conduction velocity was reduced and motor function was impaired. This demonstrates a functional relationship between internodal distance and conduction speed. Moreover, as internodes lengthened during postnatal growth, conduction velocities recovered to normal values and mutant mice exhibited normal motor and sensory behavior. This restoration of function confirms a further prediction by Huxley and Stämpfli that conduction speeds should increase as internodal distances lengthen until a “flat maximum” is reached, beyond which no further gains in conduction velocity accrue [6].

## Highlights

► Mice expressed the Schwann cell protein periaxin lacking its N-terminal PDZ domain ► Myelination was normal, but elongation of Schwann cells was retarded ► Conduction velocities were abnormally slow but recovered as internodes lengthened ► This shows that conduction velocities in myelinated nerves reach a “flat maximum”

## Results and Discussion

Huxley and Stämpfli proposed in 1949 that conduction velocities in myelinated nerves should increase with internodal distance until a “flat maximum” is reached [6]; theoretical models have tended to support this view [1–3]. However, the relationship between the distance between nodes of Ranvier and the speed of nerve conduction has not been settled experimentally [3]. The potential sensitivity of conduction speed to internodal length has important implications for nervous system function because the Schwann cells of peripheral nerves such as those that innervate limbs can increase in length dramatically during postnatal development, by 4-fold in mice [4]. Furthermore, regenerated and remyelinated peripheral nerves in rodents have uniformly short internodal lengths [5]. We have previously shown in a mouse model of the human recessive Charcot-Marie-Tooth disease 4F that Schwann cell elongation is highly retarded and that peripheral nerve conduction velocities are severely depressed [4, 7–9]. Periaxin is a component of a complex with Drp2 and dystroglycan that assembles appositions between the abaxonal surface of the myelin sheath and the Schwann cell plasma membrane, thus creating cytoplasm-filled channels called Cajal bands beneath the surface of the plasma membrane [4, 9]. Periaxin-null peripheral nerves have disrupted Cajal bands, and their Schwann cells have an impaired ability to elongate. In contrast, loss of Drp2, though it also disrupts Cajal bands, does not affect Schwann cell growth [10]. However, further study of the relationship between internodal length and conduction velocity in periaxin-null mice is not possible due to the onset of a severe demyelinating neuropathy that is also characteristic of the human disease [8, 11]. Hence, we wished to generate mice expressing a form of periaxin that affected Schwann cell elongation without the other major deleterious effects on peripheral nerve myelination observed in periaxin-null mice. Here we have taken advantage of a periaxin mutant with a highly ameliorated phenotype to examine the relationship between internodal lengths and conduction velocities.

### Generation of Mice Expressing ΔPDZ-Prx in Schwann Cells

We wished to uncouple the role of periaxin in regulating Schwann cell elongation from the severe morphological and functional consequences of its complete absence. With this goal, we generated several mouse lines expressing mutant periaxins. In the mutant described here, the N-terminal PDZ domain of periaxin was deleted. This domain homodimerizes and has been predicted to play a role, among others, in forming appositions and Cajal bands [12].

**Figure 1:**
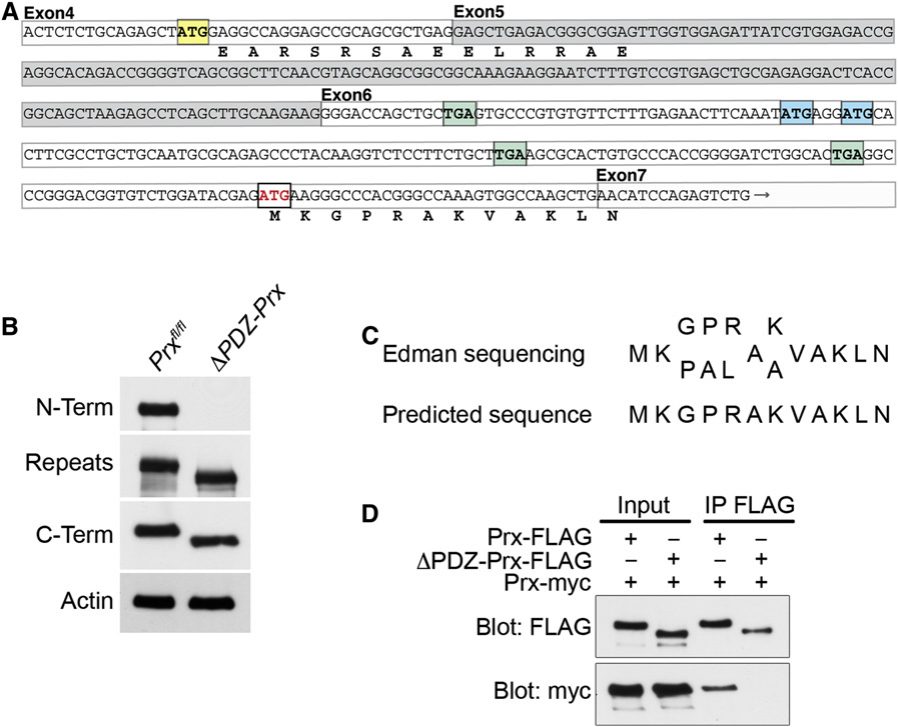
Generation of Δ*PDZ-Prx* Mice Expressing a Mutant Form of Periaxin Lacking the N-Terminal PDZ Domain (A) The exon structure that encodes the N terminus of periaxin is shown with the normal initiation codon in exon 4 highlighted in yellow. Exon 5, which is deleted after Cre-mediated recombination, is outlined in gray, and the first in-frame stop codon in exon 6 is shown in green followed by two potential initiation codons in blue. These are followed by two in-frame stop codons in green, after which the putative initiation codon utilized in Δ*PDZ-Prx* mice is highlighted in red. The amino acid sequence recognized by the N-Term anti-periaxin antibody is shown (EARSRSAEELRRAE), as is the putative N-terminal amino acid of the ΔPDZ-Prx protein (MKGPRAKVAKLN). (B) Western blot showing that an antibody raised against the peptide EARSRSAEELRRAE at the N terminus of wild-type periaxin (N-Term) does not recognize the ΔPDZ-Prx protein in extracts of sciatic nerves from 4-week-old mice, although the mutant protein reacts with two antibodies (Repeats and C-Term) that were raised against peptides encoded by exon 7. The shift to an increased mobility was also consistent with the mutant protein being slightly smaller than wild-type periaxin. γ-actin was the loading control. (C) Although there was some ambiguity at four positions, sequential amino acid sequencing of the purified ΔPDZ-Prx protein from the N terminus by the Edman degradation technique for 12 rounds confirmed the new N terminus of the ΔPDZ-Prx protein depicted in (A). (D) Coimmunoprecipitation from lysates of HEK293 cells transfected with cDNAs encoding myc-tagged wild-type periaxin with either FLAG-tagged wild-type periaxin or the myc-tagged mutant ΔPDZ-Prx showed that the mutant protein lacking the N-terminal PDZ domain did not interact with wild-type periaxin.

To obtain Δ*PDZ-Prx* mice expressing periaxin lacking the N-terminal PDZ domain, we first generated mice with *Prx* alleles in which *loxP* sequences flanked exon 5 (*Prx*^*fl/fl*^) (see Figure S1A available online). These are referred to hereafter as controls. The normal initiation codon is in exon 4 of the *Prx* gene, and the N-terminal PDZ domain is encoded by exons 5 and 6 [12]. Cre-mediated recombination of exon 5 is predicted to introduce an in-frame stop codon close to the 5′ end of exon 6; hence, translation of the periaxin mRNA ought to be terminated (Figure 1A). However, previous studies have shown that ribosomal subunits can continue to scan for a downstream initiation codon in a favorable Kozak context [13–15]. The recombined *Prx* gene sequence lacking exon 5 reveals three potential initiation codons in exon 6 (Figure 1A), but downstream of the first two of these are two in-frame termination codons (Figure 1A). Nevertheless, the third initiation codon (in red) in exon 6 is in a strong Kozak context, is in frame, and is predicted to be a translation restart site [15–17]. We speculated that translation reinitiated at this site would give rise to a protein that lacked the first 116 amino acids at the N terminus, including the PDZ domain (amino acids 13–97) [12].

Genomic PCR showed that Cre-mediated recombination using *Cnp-Cre* mice was very efficient in sciatic nerve (Figure S1B). Western blot analysis of sciatic nerve lysates revealed a mutant protein expressed at levels similar to the wild-type protein and with a size consistent with the absence of the extreme N terminus of wild-type periaxin (Figure 1B), which was confirmed using an antipeptide antibody, N-Term (Figure 1B) [12]. In contrast, the putative ΔPDZ-Prx protein was readily detectable using antibodies directed against a peptide comprising amino acids 713–728 (Repeats) [18] and a peptide sequence at the C terminus of the protein, amino acids 1350–1369 (C-Term) [9] (Figure 1B). The translational initiation site and the amino acid sequence of the novel N terminus of ΔPDZ-Prx, the mutant protein, were confirmed by sequential sequencing from the N terminus by automated Edman degradation [19]. The absence of a functional N-terminal PDZ domain in ΔPDZ-Prx was further confirmed by the inability of the mutant protein to dimerize with wild-type periaxin (Figure 1D).

### Δ*PDZ-Prx* Peripheral Nerves Have a Highly Ameliorated Phenotype Compared to Periaxin Nulls

**Figure 2:**
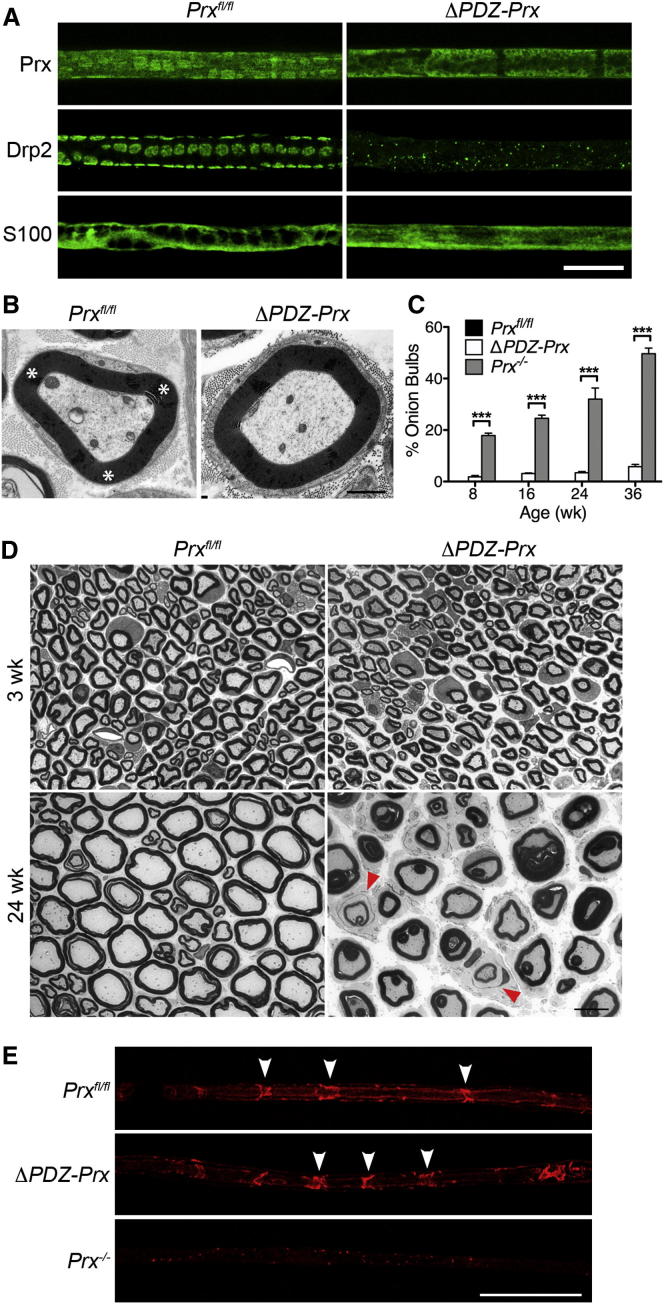
Δ*PDZ-Prx* Peripheral Nerves Have an Ameliorated Phenotype Compared to Periaxin Nulls (A) Teased fibers from control and mutant quadriceps nerves were stained by immunofluorescence with antibodies directed at periaxin (Prx), Drp2, and the cytoplasmic marker S100. Periaxin- and Drp2-stained appositions are disrupted in the mutant, as are the Cajal bands delineated by S100 staining. Scale bar represents 20 μm. (B) Electron microscopy of transverse sections from control and mutant quadriceps nerves showing the presence of appositions (asterisks) in control but their absence in mutant myelinated fibers, resulting in a concentric ring of cytoplasm around the myelin sheath. Scale bar represents 1 μm. (C) Onion bulb formations are much less abundant in Δ*PDZ-Prx* or wild-type nerves compared to periaxin-null (*Prx*^*−/−*^) nerves at all ages examined (mean values ± SEM, n ≥ 3; ^∗∗∗^p < 0.001). (D) Comparison of semithin cross sections of quadriceps nerves from control and Δ*PDZ-Prx* mice at 3 and 24 weeks. At 3 weeks, Δ*PDZ-Prx* nerves appear normal, but by 24 weeks, there are numerous nerve fibers with myelin foldings. However, onion bulb structures with thin myelin, indicative of demyelination and remyelination, are infrequent (red arrowheads). Onion bulb structures were not detectable in control nerves. Scale bar represents 10 μm. (E) Teased fibers from quadriceps nerves of 8-week-old mice were stained with fluorescent phalloidin to detect Schmidt-Lanterman incisures. Δ*PDZ-Prx* fibers had incisures that were morphologically similar to those in the control (arrowheads). In contrast, incisures were completely deranged in *Prx*^*−/−*^ nerves. Scale bar represents 50 μm.

Immunofluorescence analysis of Δ*PDZ-Prx* peripheral nerves showed that the PDZ domain is required for the formation of domains enriched in Drp2-periaxin-dystroglycan complexes in the plasma membrane of Schwann cells, and loss of appositions was confirmed by electron microscopy (Figure 2B). Despite the loss of Cajal bands, myelination was unaffected and levels of the major peripheral nervous system myelin proteins P0 and myelin basic protein (MBP) were unaffected in the mutant (Figure S2A). Consistent with normal myelination, the mutant had normal g ratios at 3 weeks (0.61 ± 0.02 and 0.64 ± 0.01, mean values ± SEM, n = 3 for control and mutant respectively, p = 0.24) and normal axon diameters (2.65 ± 0.05 μm and 2.55 ± 0.09 μm, n = 3 for control and mutant respectively, p = 0.43). The distribution of myelin thickness with respect to axonal diameter was also unaffected in the mutant (Figure S2B).

Disturbances to myelination at older ages were very mild in Δ*PDZ-Prx* mice, and onion bulb profiles with supernumerary Schwann cells, an indicator of demyelination and remyelination, were much less abundant in Δ*PDZ-Prx* mice compared to periaxin nulls (Figures 2C). Interestingly, the low percentage of onion bulbs in Δ*PDZ-Prx* at 36 weeks was comparable to that in Drp2-null mice, which also have disrupted Cajal bands and a mild phenotype [10], in marked contrast to periaxin-null nerves at the same age (Figure 2D). Also similar to Drp2-null nerves, there was no decrease in the number of Schmidt-Lanterman incisures in Δ*PDZ-Prx* mice measured at 8 weeks; in fact, there was a modest increase (2.20 ± 0.04 per 100 μm and 2.63 ± 0.09 per 100 μm, n = 4 for control and Δ*PDZ-Prx* mice respectively, p < 0.05) (Figure 2E) [10]. In contrast, and as we have observed previously [8], Schmidt-Lanterman incisures were barely detectable in periaxin-null nerves (Figure 2E), again underlining the ameliorated nature of the phenotype in Δ*PDZ-Prx* mice. Other key structural features that could affect nerve function include the organization of the nodal, paranodal, and juxtaparanodal domains, but these were unaffected in the mutant (Figures S2C and S2D).

Because disruption of Cajal bands per se does not influence either Schwann cell elongation or conduction velocity [10], the Δ*PDZ-Prx* mice appeared to be excellent subjects in which to investigate how internodal lengths influence conduction velocity during postnatal development through to maturity.

### Reduced Schwann Cell Elongation in Δ*PDZ-Prx* Mice Retards but Does Not Prevent Development of Normal Conduction Velocities to a Maximum Value

**Figure 3:**
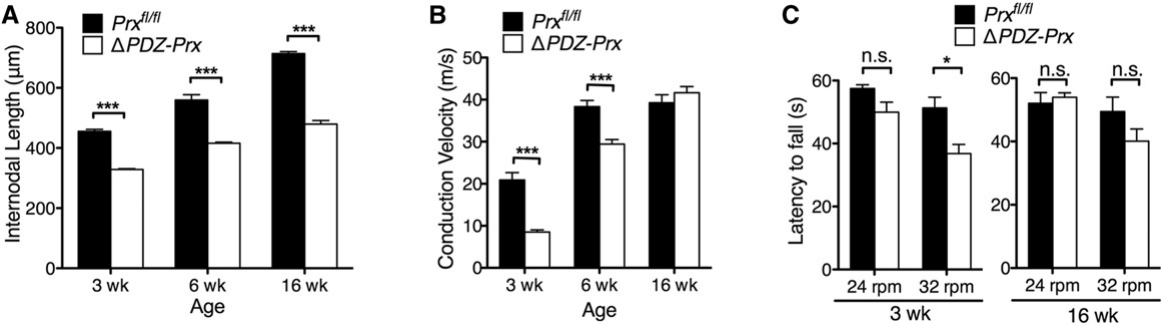
Δ*PDZ-Prx* Mice Recover Normal Peripheral Nerve Conduction Velocities and Behavior after Retarded Schwann Cell Growth during Development (A) Internodal lengths of teased quadriceps fibers from control and Δ*PDZ-Prx* nerves at 3, 6, and 16 weeks (mean values ± SEM, n ≥ 5 per group; ^∗∗∗^p < 0.001). (B) Nerve conduction velocities in control and Δ*PDZ-Prx* quadriceps nerves at 3, 6, and 16 weeks (mean values ± SEM, n ≥ 5 per group; ^∗∗∗^p < 0.0001). Conduction velocities at 16 weeks in Δ*PDZ-Prx* nerves were not significantly different from controls. (C) Motor coordination was evaluated in control and Δ*PDZ-Prx* mice at 3 and 16 weeks using the rotarod test. At 24 rpm, there was no difference in rotarod performance between control and Δ*PDZ-Prx* mice at both ages. However, under more demanding conditions at 32 rpm, Δ*PDZ-Prx* mice performed significantly worse (mean values ± SEM, n ≥ 12 per group; n.s., not significant, ^∗^p < 0.05). Motor coordination of 16-week-old Δ*PDZ-Prx* mice did not differ significantly from control values at 32 rpm.

We have shown previously that the absence of periaxin inhibits Schwann cell elongation when nerves are lengthening during limb growth [4]. When wild-type periaxin was replaced by the ΔPDZ-Prx protein at an early stage of postnatal development, Schwann cell elongation was similarly compromised (Figure 3A). Consistent with the shorter internodal lengths, there was a proportionate increase in the number of Krox-20- and Sox10-positive Schwann cells (Figure S3). Reduced internodal lengths were reflected at 3 weeks in a >50% reduction in conduction velocity in the mutant (Figure 3B), at a time when g ratios and axon diameters were normal (see above). The conduction velocities of Δ*PDZ-Prx* mice were similarly depressed compared to periaxin nulls at 3 weeks (8.5 ± 0.5 ms^−1^ and 12.6 ± 1.1 ms^−1^, Δ*PDZ-Prx* and *Prx*^*−/−*^ mice respectively, n ≥ 8, p = not significant), although we found a small difference in their abnormally short internodal lengths (328.7 ± 2.4 μm and 302.7 ± 3.9 μm, Δ*PDZ-Prx* and *Prx*^*−/−*^ mice respectively, n ≥ 5, p < 0.05). This suggests that the PDZ domain in periaxin has a determining role in the ability of this protein to regulate Schwann cell elongation in addition to its role in the formation of appositions.

As the distance between nodes grew in the mutant nerves, their conduction velocities increased (Figure 3B). However, by 6 weeks, control nerves had reached their maximum conduction velocity of around 40 ms^−1^ (Figure 3B), whereas the conduction velocity in mutant nerves was still retarded. Nevertheless, by 16 weeks, the speed of conduction in mutants had caught up with and was indistinguishable from controls (Figure 3B). The attainment of normal nerve conduction by 16 weeks in the mutants was reflected in the restoration of normal motor performance in the rotarod test (Figure 3C). Sensory tests were also normal at this age using the hindpaw withdrawal response after mechanical stimulation (162.8 ± 4.2 mN/mm^2^ and 178.3 ± 9.6 mN/mm^2^, control and Δ*PDZ-Prx* mice respectively, n ≥ 9, p = 0.22) or withdrawal latency from noxious thermal stimulation (7.1 ± 0.5 s and 7.2 ± 0.6 s, control and Δ*PDZ-Prx* mice respectively, n ≥ 9, p = 0.96). Although Δ*PDZ-Prx* mice had recovered normal conduction speeds in their quadriceps nerves by 16 weeks (Figure 3B), periaxin-null nerves still displayed highly depressed rates of conduction (41.6 ± 1.5 ms^−1^ and 17.9 ± 1.9 ms^−1^, Δ*PDZ-Prx* and *Prx*^*−/−*^ mice respectively, n ≥ 6, p < 0.0001). The extensive demyelination that afflicts the peripheral nervous system of periaxin-null mice likely affects their ability to recover normal conduction speeds even though their internodal lengths increase. Hence, although delayed, it appears that the peripheral nerves of Δ*PDZ-Prx* mice attain normal speeds of nerve impulse conduction and normal motor and sensory function once the distance between nodes has reached the threshold of the flat maximum [6].

### Conclusions

This study on the relationship between internodal length and conduction velocity in myelinated peripheral nerves exploited a mouse whose Schwann cells express a mutant periaxin protein that does not cause severe demyelination yet still retains the retarded elongation phenotype of the periaxin-null mouse [9, 18]. Computer simulations of conduction in myelinated nerve fibers have suggested that nerve conduction velocities should be sensitive to internodal length in the shorter range but become less sensitive at longer Schwann cell lengths [20, 21]. An earlier study of nerve conduction velocity in regenerated peripheral fibers showed that although axons regained normal diameters and myelin sheath thickness, their internodal lengths were reduced; nevertheless, nerve conduction velocities were normal [22]. Based on their theoretical analysis, Huxley and Stämpfli proposed that this was because the internodal lengths of regenerated fibers had reached the range where conduction velocity would no longer be sensitive to internodal length [6]. As these authors noted, “We should expect the difference in velocity (between control and regenerated fibers) to be least if the normal spacing were somewhat above, and the reduced spacing below, the value which would give maximum velocity.” They also pointed out the evolutionary significance of this prediction in that natural selection is likely to have resulted in nodal spacing in mature nerves that permits significant deviations in internodal length without affecting conduction velocity.

This exposition is persuasive because, although values for internodal lengths were not quoted by Sanders and Whitteridge [22], it can be inferred from their Figure 11 that internodal lengths in rabbit peroneal nerves were still in excess of 800 μm after axon regeneration. This is in marked contrast to the range of internodal lengths studied in the Δ*PDZ-Prx* mutant (329 to 479 μm, from 3 weeks to 16 weeks respectively). Theoretical considerations would suggest that the internodal lengths in the regeneration study [22] would be within the flat maximum [1, 4]. Similarly, the acquisition of normal rates of nerve conduction in older Δ*PDZ-Prx* nerves appears to be a result of Schwann cells having elongated sufficiently to allow for optimal conduction, together with the normal increase in axon diameter and myelin thickness that occurs during development. This supports the view that the internodal lengths of older Δ*PDZ-Prx* Schwann cells lie in the range in which mathematical models predict the flat maximum of Huxley and Stämpfli [6].

We have shown that the velocity of nerve impulse conduction in myelinated nerves is determined by the distance between nodes of Ranvier until a threshold of internodal distance is reached, beyond which conduction rates plateau. The fact that normal Schwann cells in murine peripheral nerves reach this maximum conduction speed before they have reached their maximum length suggests that there is a considerable safety factor to ensure that myelinated axons conduct optimally. Furthermore, a delay in reaching these maxima in mutant nerves appears to have no adverse effect on mature motor and sensory behavior, showing that derangements to Schwann cell growth need not ultimately compromise peripheral nerve function.

## Experimental Procedures

### Generation of Δ*PDZ-Prx* Mice

All animal work conformed to the UK Animals (Scientific Procedures) Act 1986 and to University of Edinburgh Ethical Review Committee policy. *Cnp1-Cre* mice have been described previously and shown to be effective in promoting Cre-mediated recombination in mouse embryonic peripheral nerves before the radial sorting of axons [23–25]. Mice carrying a *Prx* floxed allele were generated by homologous recombination as described in Supplemental Experimental Procedures. Mice expressing mutant periaxin in Schwann cells were generated by crossing mice carrying floxed alleles of *Prx* with *Cnp1-Cre* mice [24].

### cDNA Constructs and Transfection

Generation of rat full-length periaxin cDNA (nt 268–4421; aa 1–1384) in the mammalian expression vectors pFLAG-CMV5a (Sigma) with an introduced XbaI site and pCB6myc (gift of D. Russell, University of Texas) was performed as described previously [7]. The constructs were expressed as full-length periaxin with a C-terminal myc epitope tag or a C-terminal FLAG tag, respectively, and were used as positive controls. Generation of ΔPDZ-Prx cDNAs and transfections are described in Supplemental Experimental Procedures.

### Immunostaining, Western Blotting, and Histology

All histology and immunofluorescence analyses were performed on quadriceps nerves unless specified otherwise. The perineurium was removed prior to immunostaining of teased fibers. Further preparation and the method for immunostaining of cryosections or teased fiber preparations were as described previously, and all primary and secondary antibodies and nuclear stains have been described previously [4, 7]. Conventional and confocal fluorescence, electron microscopy, and western blotting of sciatic nerve lysates were performed as described in Supplemental Experimental Procedures. Light micrographs were of toluidine blue-stained transverse sections of quadriceps nerve.

### Electrophysiology and Behavioral Testing

Acutely prepared quadriceps nerves from control and mutant mice were placed in oxygenated mammalian HEPES physiological solution (137 mM NaCl, 5 mM KCl, 2 mM CaCl_2_, 1 mM MgCl_2_, 5.5 mM D-glucose, 5 mM HEPES [pH 7.2–7.4]). Nerve conduction velocities were measured as described previously [4]. Fixed-speed rotarod analysis and sensory reflex testing were performed as described previously [4, 26].

### Edman Degradation

In order to determine the N-terminal sequence of the mutant ΔPDZ-Prx protein by Edman degradation, we purified the protein using a GST-fusion protein containing the third fibronectin III domain of β4-integrin, which strongly interacts with the C terminus of periaxin. Briefly, the β4-integrin third fibronectin III was amplified by PCR and subcloned in frame into pGEX-KG for generation of GST fusion proteins. GST fusion constructs were overexpressed in *E. coli* and purified as described previously [7]. The mutant protein was purified by SDS-PAGE, and the N terminus was then sequenced by the Edman degradation method (Aberdeen Proteomics, University of Aberdeen).

### Morphometry

The diameters of axons, axons plus myelin, the resulting g ratios, and internodal lengths were calculated from a minimum of 100 axons per animal, and a minimum of 3 animals were used per condition as described previously [4, 25]. Schmidt-Lanterman incisures were visualized with Alexa Fluor 568-phalloidin.

### Statistical Analysis

Statistical analysis was performed by unpaired Student’s t test (unless specified otherwise) or by ANOVA with GraphPad Prism 5.0c software.

## Supplemental Information

Document S1. Figures S1–S3 and Supplemental Experimental Procedures
