# Supplementary material for: Increasing Internodal Distance in Myelinated Nerves Accelerates Nerve Conduction to a Flat Maximum
Source: Curr Biol. 2012 Oct 23;22(20):1957–61. doi: 10.1016/j.cub.2012.08.025 (PMC3482659; doi:10.1016/j.cub.2012.08.025)
Supplement: Document S1. Figures S1–S3 and Supplemental Experimental Procedures [file mmc1.pdf]

Current Biology, Volume *22*

Supplemental Information

Increasing Internodal Distance in Myelinated Nerves Accelerates Nerve Conduction to a Flat Maximum

Lai Man N. Wu, Anna Williams, Ada Delaney, Diane L. Sherman, and Peter J. Brophy

Supplemental Inventory

Figure S1
Shows the targeting strategy and demonstration of successful recombination to generate the mice used in this study.

Figure S2
Comprises a Western blot that confirms that myelination has been unaffected in the mutant, a plot of myelin thickness versus axon diameter showing that the distribution of myelin thickness with respect to axon diameter is unchanged in the mutant and electron micrograph and immunofluorescence images showing that the structure of the node, paranode and juxtaparanode are normal in the mutant.

Figure S3
Validates the reductions in the lengths of individual Schwann cells by showing that the number of myelinating Schwann cells in mutant nerves is increased.

Supplemental Experimental Procedures

Supplemental References

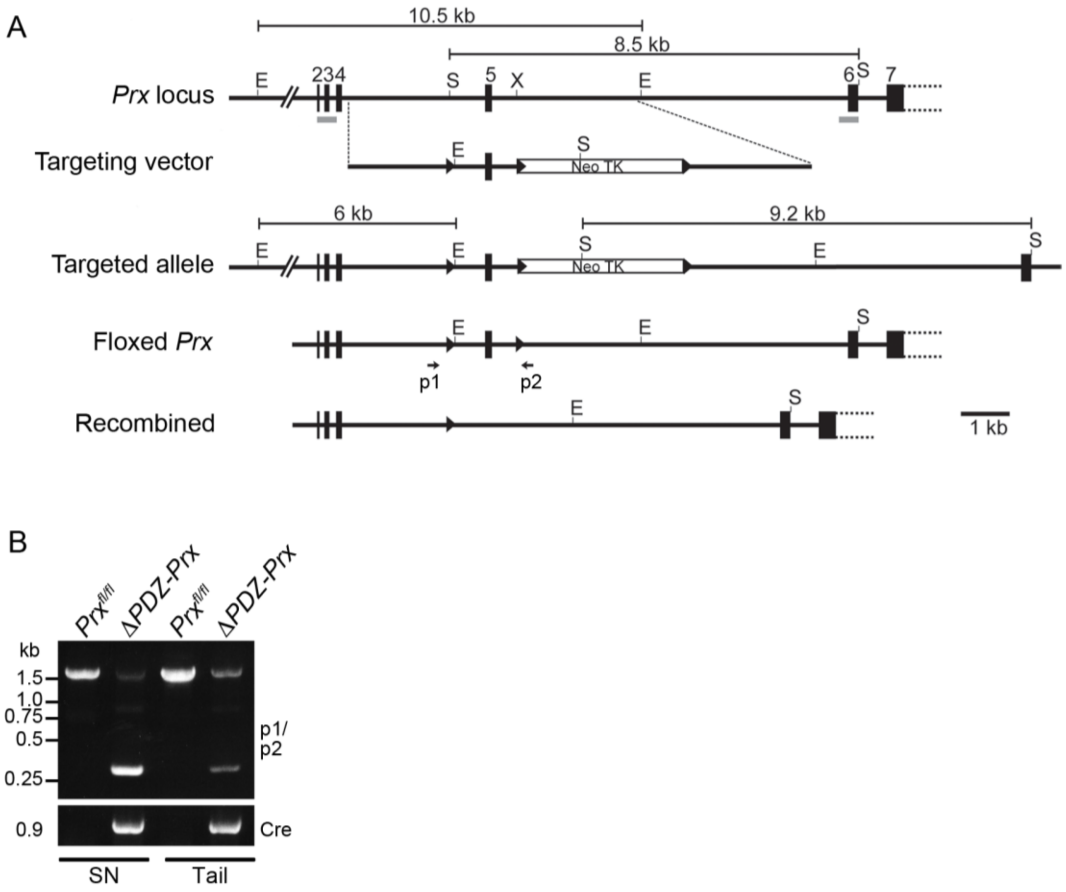

Figure S1. Targeting Strategy and PCR Demonstrating Successful Cre-Mediated Recombination in Sciatic Nerve (Related to Figure 1)

(A) Schematic of the *Periaxin* gene (*Prx*), with targeting vector, floxed, and mutant recombined loci. Two *lox*P (▶) sites were positioned to flank exon 5 with a third at the 3′ end of the Neo selection cassette. The cassette was removed by Cre transfection of targeted embryonic stem cells. The *loxP* sites (black triangles) are indicated. E: EcoRI sites; S: SacI sites. P1 and p2 indicate the positions of primers used for genotyping.

(B) Recombination in $Prx^{fl/fl}$/*Cnp1*-$Cre^{+/-}$ mice was detected by genomic PCR using primers p1 and p2. PCR using Cre-specific primers detected Cre. SN: sciatic nerve genomic DNA; Tail: tail genomic DNA.

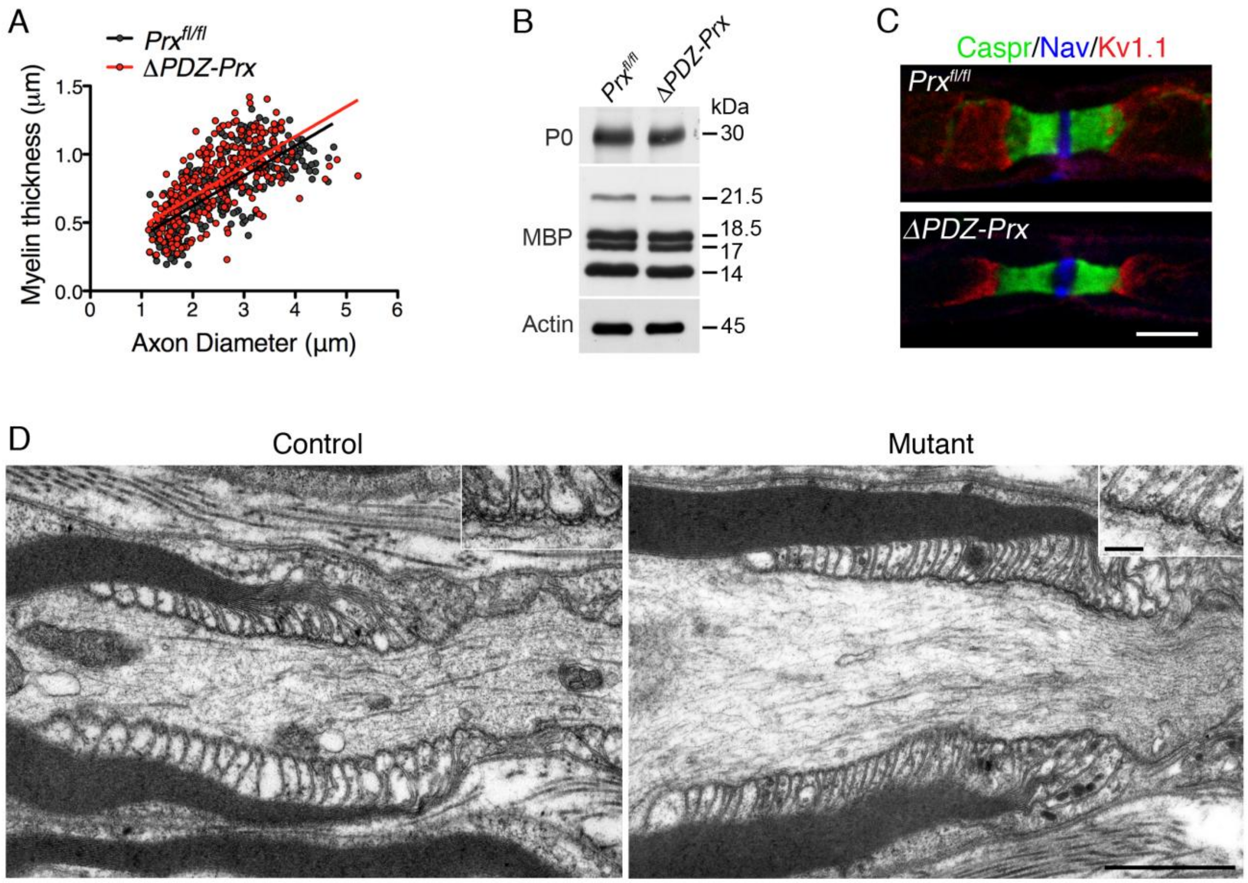

Figure S2. Major Myelin Proteins, Myelin Thickness, and Structural Organization of the Node Are Unaffected in the Mutant (Related to Figure 2)

(A) The slopes of the regression lines for myelin thickness versus axonal diameter were not significantly difference between control and mutant showing that the size distribution of myelinated axons was unaffected (slopes are 0.221 and 0.219 for control and mutant respectively, $p = 0.92$).

(B) Western blot of P_0 and Myelin Basic protein (MBP) of sciatic nerve homogenates from control and *ΔPDZ-Prx* mice at 8 weeks showed normal levels in the mutant. γ-Actin was the loading control and molecular weights of standard proteins in kilodaltons (kDa) are indicated on the right.

(C) Immunofluorescence staining of quadriceps nerves at P21 shows normal localization of sodium channels (Nav) at the node (blue), Caspr at the paranodes (green) and potassium channels (Kv1.1) at the juxtaparanodes (red). Scale bar represents 5 µm.

(D) Electron micrographs of longitudinal sections of quadriceps nerves at P21 in the vicinity of the node show normal morphology of nodes and paranodes in the mutant. Scale bar represents 1 µm. The insets show the presence of septate junctions at the paranode in both control and mutant. Scale bar represents 0.1 µm.

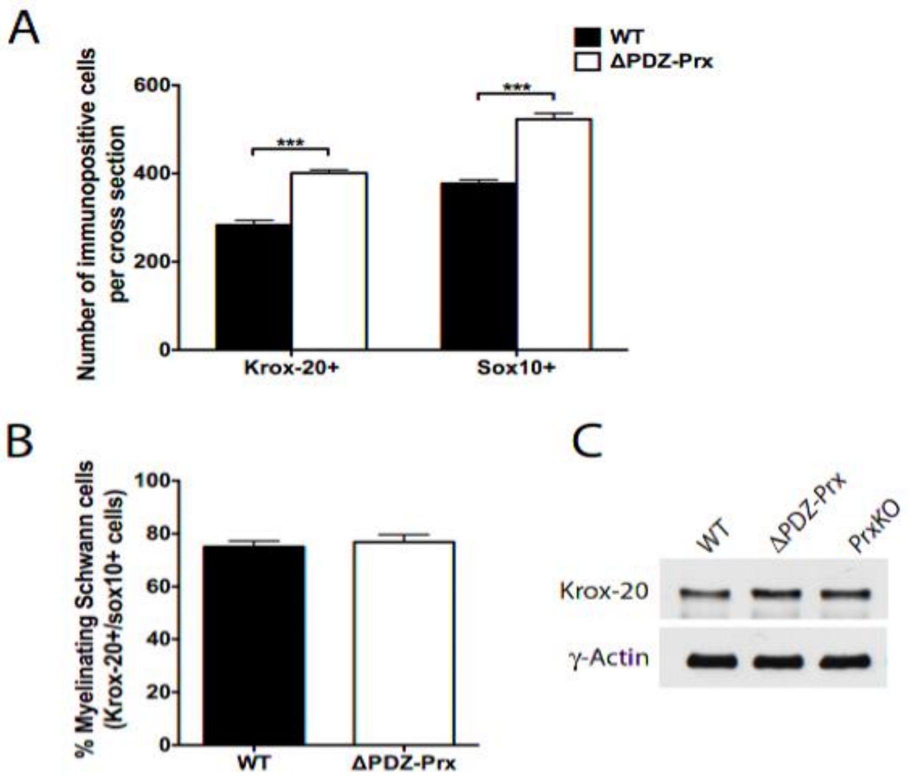

Figure S3. An Increase in Schwann Cell Number Is Consistent with a Decrease in Internodal Length in *ΔPDZ-Prx* Peripheral Nerves (Related to Figure 3)

(A) Sciatic nerve transverse sections from 3-week-old control (WT) and *ΔPDZ-Prx* mice were immunolabeled for Krox-20 and Sox10 (myelinating and total Schwann cells respectively). Mean values (± SEM, n = 3) for both Krox-20+ and Sox10+ cells were significantly higher in the mutant (both $p < 0.001$).

(B) The proportion of myelinating Schwann cells (ratio of Krox-20+ to Sox10+ cells) was also unaffected in mutant sciatic nerves (mean values ± SEM, p not significant)).

(C) Western blot shows corresponding raised levels of Krox-20 in mutant sciatic nerves at 3 weeks. γ-Actin was the loading control.

Supplemental Experimental Procedures

Generation of *ΔPDZ-Prx* Mice

All animal work conformed to UK legislation (Scientific Procedures) Act 1986, and to the University of Edinburgh Ethical Review Committee policy. *Cnp1-Cre* mice have been described and shown to be effective in promoting Cre-mediated recombination in mouse embryonic peripheral nerves before the radial sorting of axons [1-3]. The *Prx* gene contains 7 exons, of which exons 4-7 are coding exons. 91 % of Periaxin is encoded by exon 7, whereas the PDZ domain is encoded by both exons 5 and 6. Mice carrying a *Prx* floxed allele were generated by homologous recombination in E14-TG2a.IV (129/Ola strain) ES cells by methods described previously [4]. The strategy used for gene targeting is summarized in Figure S1. The replacement targeting vector contained three *loxP* sequences in the same orientation. The first *loxP* sequence was inserted at a *Sma*I site in intron 4, 714 bp upstream of exon 5, and the other *loxP* sequences were flanking a PGK*neo*-HSV*tk* positive-negative selection cassette (gift of Dr Andrew Smith, University of Edinburgh) which was introduced at an *Xba*I site in intron 5 located 519 bp downstream of the end of exon 5. For the initial targeting, ES cells were selected in G418 only and resistant clones screened by correct targeting was confirmed by Southern blot analysis of EcoR I digested genomic DNA by hybridization with a 5´ probe external to the homology region and using a probe 3´ to the homology region with Sma I digested DNA. The 5´ probe detected a 10.5 kb fragment corresponding to the wild type allele and a 6 kb fragment corresponding to the targeted allele and the 3´ probe detected a 8.5 kb fragment corresponding to the wild type allele, and an 9.2 kb fragment corresponding to the targeted allele. A targeted clone was expanded in nonselective medium and transfected with Cre recombinase-expresion plasmid pCAGGS-Cre-IRES*puro* to generate cells with a deletion of the selection cassette. Transfected cells were selected with 2.5 μM ganciclovir and genomic DNA from resistant clones were screened by Southern blot analysis. Two correctly-targeted clones were injected into blastocysts. Both clones gave good germ line transmitting chimeras in test-crosses with the C57BL/6 strain and offspring heterozygous for the correctly-targeted allele were subsequently identified by ear biopsy and PCR analysis. Heterozygotes were then back-crossed onto the C57BL/6 background for at least 10 generations prior to experimental analysis. Genotyping of $Prx^{fl/fl}$/$Cnp1$-$Cre^{+/-}$ mice by successive PCRs of genomic DNA extracted from ear biopsies was using the following primers, first 5′-CCTGTGACTTCCCAAAAAAAC-3′ and 5′ AAGAAGAGGGGATGAGTAGGCG-3′ yielding a 1.0 kb product which after *EcoR* I digestion yielded a 0.5 kb fragment for the floxed allele but remained undigested for the wild-type allele. Secondly, the presence of *Cre* was detected as described [2].

cDNA Constructs and Transfection

Generation of rat full length Periaxin cDNA (nt 268-4421; aa 1-1384) in the mammalian expression vectors pFLAG-CMV5a (Sigma) with an introduced XbaI site and pCB6myc (gift of D. Russell, University of Texas) was performed as previously described [5]. The constructs were expressed as full length Periaxin with a C-terminal myc-epitope tag or a C-terminal FLAG tag, respectively, and were used as positive controls. To generate ΔPDZ-Prx (nt 618-4421; aa 116-1384) in pFLAG-CMV5a, a BglII site was introduced downstream of the PDZ domain sequence using rat Periaxin cDNA in pSPORT. The PCR product was purified, restriction digested with BglII and KpnI, and cloned into BglII/KpnI digested Peri-pSP72. Correct sequence of the PCR generated region in the construct was confirmed by DNA sequencing. ΔPDZ-Prx cDNA was

excised from pSP72 by BglII and XbaI, and cloned into BglII/XbaI digested pFLAG-CMV5a. Transfections were using FuGENE 6 (Roche) in HEK 293 cells. 48 h following transfection, cells were washed once with cold PBS, scraped off the dish and lysed with IP buffer (50 mM Tris pH 7.5, 150 mM NaCl, 1% Triton X-100, 1 mM EDTA, and phosphatase and proteinase inhibitors: 5 mM NaF, 1 mM Na_3VO_4, 2 mM $Na_4P_2O_7$, 0.5 mM TLCK, 1 mM benzamidine, 1 mM PMSF, 10 µg/ml leupeptin, 10 µg/ml antipain) for 30 min at 4°C. Following centrifugation at 13000 rpm for 20 min at 4°C, 1/10 of the lysate was set aside as input and the rest was incubated with 16 µl washed anti-FLAG M2 affinity gel (Sigma) at 4°C overnight on wheel. Next day, the coupled FLAG M2 beads were pelleted by centrifugation at 6000 rpm, and washed 6-10 times with IP buffer and once with the same buffer without detergent. Immunoprecipitated proteins were eluted with SDS-PAGE sample buffer and resolved on 5.5% SDS polyacrylamide gels. Protein interaction was analysed by Western blotting using anti-FLAG M2 monoclonal antibody (1:8000, Sigma) and anti-c-myc (1:10, 9E10, Sigma). The experiment was performed at least three times.

Immunostaining, Western Blotting, and Histology

All histology and immunofluorescence analyses were performed on quadriceps nerves unless specified otherwise. The perineurium was removed prior to immunostaining of teased fibers. Further preparation and the method for immunostaining of cryosections or teased fiber preparations were as described previously and all primary and secondary antibodies and nuclear stains have been described [5, 6]. Conventional microscopy was performed using an Olympus BX60 microscope and images were captured using a Hamamatsu Orca-ER camera and Improvision Openlab software. For confocal microscopy we used a Leica TCL-SL confocal microscope with either a 40x or a 63x objective, 1.4 NA, and Leica proprietary software. The acquired stacks were assembled using the maximum projection tool and all figures were prepared using Adobe Photoshop CS4 extended version 11 and were not subjected to any subsequent image processing. Western blotting was performed as described on sciatic nerve lysates [4]. prepared in 1% NP-40, 0.5% sodium deoxycholate, 0.1% SDS, 50 mM Trs-HCl pH7.5, 150 mM NaCl, 1 mM EDTA, 1 mM sodium orthovanadate, 2 mM sodium pyrophosphate, 10 mM NaF with proteinase inhibitors, 1 mM benzamidine, 0.5 mM TLCK, 1 mM PMSF and leupeptin and antipain, both 10 mg/ml. For electron microscopy mice were perfused intravascularly with 2.5% glutaraldehyde and 4% paraformaldehyde in 0.1 M sodium cacodylate buffer (pH 7.4). Nerves were removed, fixed for 2 h at room temperature then 18 h at 4°C in the same fixative, postfixed in OsO4, and embedded in araldite. Ultrathin sections were stained with uranyl acetate and lead citrate and examined on a Phillips BioTwin CM120 electron microscope equipped with a Gatan Orius camera.

Supplemental References

1. Grove, M., Komiyama, N.H., Nave, K.A., Grant, S.G., Sherman, D.L., and Brophy, P.J. (2007). FAK is required for axonal sorting by Schwann cells. J. Cell Biol. *176*, 277-282.
2. Lappe-Siefke, C., Goebbels, S., Gravel, M., Nicksch, E., Lee, J., Braun, P.E., Griffiths, I.R., and Nave, K.A. (2003). Disruption of Cnp1 uncouples oligodendroglial functions in axonal support and myelination. Nat. Genet. *33*, 366-374.
3. Sherman, D.L., Krols, M., Wu, L.M., Grove, M., Nave, K.A., Gangloff, Y.G., and Brophy, P.J. (2012). Arrest of myelination and reduced axon growth when Schwann cells lack mTOR. J. Neurosci. *32*, 1817-1825.
4. Sherman, D.L., Tait, S., Melrose, S., Johnson, R., Zonta, B., Court, F.A., Macklin, W.B., Meek, S., Smith, A.J., Cottrell, D.F., et al. (2005). Neurofascins are required to establish axonal domains for saltatory conduction. Neuron *48*, 737-742.
5. Guilbot, A., Williams, A., Ravise, N., Verny, C., Brice, A., Sherman, D.L., Brophy, P.J., LeGuern, E., Delague, V., Bareil, C., et al. (2001). A mutation in periaxin is responsible for CMT4F, an autosomal recessive form of Charcot-Marie-Tooth disease. Hum. Mol. Genet. *10*, 415-421.
6. Court, F.A., Sherman, D.L., Pratt, T., Garry, E.M., Ribchester, R.R., Cottrell, D.F., Fleetwood-Walker, S.M., and Brophy, P.J. (2004). Restricted growth of Schwann cells lacking Cajal bands slows conduction in myelinated nerves. Nature *431*, 191-195.
